# Conversion of Tumors into Autologous Vaccines by Intratumoral Injection of **α**-Gal Glycolipids that Induce Anti-Gal/**α**-Gal Epitope Interaction

**DOI:** 10.1155/2011/134020

**Published:** 2011-11-17

**Authors:** Uri Galili

**Affiliations:** Department of Surgery, University of Massachusetts Medical School, 55 Lake Avenue North, Worcester, MA 01655, USA

## Abstract

Anti-Gal is the most abundant antibody in humans, constituting 1% of immunoglobulins. Anti-Gal binds specifically **α**-gal epitopes (Gal**α**1-3Gal**β**1-4GlcNAc-R). Immunogenicity of autologous tumor associated antigens (TAA) is greatly increased by manipulating tumor cells to express **α**-gal epitopes and bind anti-Gal. Glycolipids with **α**gal epitopes (**α**-gal glycolipids) injected into tumors insert into the tumor cell membrane. Anti-Gal binding to the multiple *α*-gal epitopes *de novo* presented on the tumor cells results in targeting of these cells to APC via the interaction between the Fc portion of the bound anti-Gal and Fc**γ**; receptors on APC. The APC process and present immunogenic TAA peptides and thus, effectively activate tumor specific CD4+ helper T cells and CD8+ cytotoxic T cells which destroy tumor cells in micrometastases. The induced immune response is potent enough to overcome immunosuppression by Treg cells. A phase I clinical trial indicated that **α**-gal glycolipid treatment has no adverse effects. In addition to achieving destruction of micrometastases in cancer patients with advance disease, **α**-gal glycolipid treatment may be effective as neo-adjuvant immunotherapy. Injection of **α**-gal glycolipids into primary tumors few weeks prior to resection can induce a protective immune response capable of destroying micrometastases expressing autologous TAA, long after primary tumor resection.

## 1. Introduction 

Destruction of detectable metastases by resection or ablation may prolong survival, but it does not affect invisible micrometastases which can develop into lethal metastases. Moreover, it is likely that in a large proportion of patients treated by novel targeted therapies, micrometastases may not be completely eliminated. This is since there is a high probability that some metastatic cells residing far enough from capillaries will not be susceptible to the therapeutic effect of drugs because of diminishing concentration of drugs diffusing from the nearby capillary. A lasting antitumor protection that effectively destroys micrometastases may be achieved by immunotherapy that stimulates the immune system to react against the multiple tumor-associated antigens (TAAs). This principle is demonstrated in the life-long protection against EBV transformed polyclonal B cells which are “kept at bay” by EBV-specific T cells. Immunosuppression (as in allograft recipients) may result in appearance of such transformed B cells as multiclonal lymphomas due to the suppression of protective T cell activity [1]. The protective effect of the human immune system against TAA on tumor cells is further illustrated by the high correlation between the extent of T cell infiltration within tumors and positive prognosis reported in melanoma, ovarian cancer, colorectal carcinoma, and other tumors [2–6]. In particular, detailed studies of Galon and colleagues demonstrated a distinct inverse correlation between the density of infiltrating CD8+ and Th1 memory T cells in resected colorectal carcinoma and the relapse of the disease [4–6]. The observed exclusive infiltration of T cells into tumors without affecting normal tissues implies that the observed antitumor immune response is aimed specifically against antigens (Ags) on tumor cells, that is, against TAA. This further implies that eliciting an immune response against these Ags may result in immune protection against the tumor cells expressing them.

## 2. Autologous TAA as Antitumor Vaccine

TAA can be common to a given tumor in many patients having the same type of tumor, or can be unique to the individual patient. Immunotherapy against known common TAA has been unsatisfactory, possibly since such TAA are of weak immunogenicity due to their presence in low amounts both on normal cells and on embryonic cells [7, 8]. Unique TAA appears due to genomic instability in tumor cells and are generated by multiple coding mutations which differ from one patient to the other. Many of these mutations result in small changes in proteins that may provide advantageous growth to tumor cells [9–13]. Other mutations are neutral since they do not affect the structure or function of the mutated protein. The mutated proteins can function as autologous TAA that may elicit a protective antitumor immune response, since they are present in tumor cells and absent in normal cells. Such a protective immune response against autologous TAA may be beneficial in achieving immune protection against metastatic tumor cells. The immune system is capable of detecting and reacting against small changes that give rise to autologous TAA. This can be inferred from the extensive immune response to blood group Ags where the difference of the small N-acetyl group on the terminal galactosyl in blood group A, in comparison to blood group B (i.e., GalNAc*α*1-3Gal and Gal*α*1-3Gal, resp.) is sufficient for inducing the production of anti-A antibody (Ab) in blood group B individuals. 

Characterization and production of the multiple autologous TAA in each individual patient for vaccinating purpose is not feasible at present. Thus, the tumor itself is the only current practical source for autologous TAA [14]. As with any microbial vaccine, an effective immune response against autologous TAA requires effective uptake of the tumor cells and cell membranes by antigen presenting cells (APCs) such as dendritic cells and macrophages. The internalized TAA are transported by APC to the regional lymph nodes, processed by these cells, and presented on MHC class I and class II molecules as TAA peptides that activate tumor-specific CD8+ cytotoxic T cells and CD4+ Th1 cells, respectively [15, 16]. However, vaccination with unprocessed tumor cells obtained from the patient is usually ineffective since tumor cells evolve to evade recognition by APC [7–10] and thus are “ignored” by the immune system. This is clearly indicated by the ability of tumor cells to reside in lymph nodes without being affected by the immune system. Moreover, TAA are usually “concealed” from the immune system because the tumor cytokine milieu is often suppressive toward immune function and induces tolerance, anergy, or lymphocyte death [17–19]. Thus, studies have been aimed to recruit APC into the lesions by intratumoral administration of immunomodulators such as GM-CSF or CpG oligonucleotides [19, 20]. Although effectively recruited, the APC cannot identify tumor cells within the lesion as cells that “ought” to be internalized, since the tumor cells lack identifying markers that label them for uptake by APC. Thus, uptake of tumor cells by recruited APC is suboptimal as it is mediated by random endocytosis [14]. 

It is well established that one of the most effective mechanisms by which APC as macrophages and dendritic cells can internalize vaccinating Ags and process them for T cell activation is the formation of immune complexes with the microbial and tumor vaccinating Ags. The interaction of the Fc portion of the immunocomplexed Ab molecules with Fc*γ* receptors (Fc*γ*Rs) on APC generates signals for Ag internalization as well as for maturation of dendritic cells internalizing the Ag and subsequent effective stimulation of the immune system [21–30]. Targeting tumor cells via Fc/Fc*γ*R interaction to macrophages and dendritic cells enables these APC to internalize TAA of the tumor cells coated with an IgG Ab. Such targeting is feasible in all humans that are not severely immunocompromized by exploiting the natural anti-Gal Ab and its ligand the *α*-gal epitope.

## 3. The Natural Anti-Gal Antibody and The ***α***-gal Epitope

Anti-Gal is the most abundant natural Ab in human blood, constituting ~1% of serum immunoglobulins [31]. It is produced throughout life in response to antigenic stimulation by gastrointestinal bacteria [32]. Anti-Gal binds specifically to the *α*-gal epitope. This epitope has the structure Gal*α*1-3Gal*β*1-4GlcNAc-R [33, 34]. The *α*-gal epitope is present on cell surface glycolipids and glycoproteins of nonprimate mammals, prosimians, and New World monkeys [35–37]. The *α*-gal epitope is synthesized on carbohydrate chains of glycolipids and glycoproteins in mammalian cells by the glycosylation enzyme *α*1,3galactosyltransferase (*α*1,3GT) [36, 38]. Because of immune tolerance, mammalian species producing *α*-gal epitopes lack the natural anti-Gal Ab. In contrast, humans, apes, and Old World monkeys lack the *α*-gal epitope due to inactivation of the *α*1,3GT (*Ggta1*) gene in ancestral primates, and all produce the natural anti-Gal antibody [35–41]. 

Since anti-Gal is present in large amounts in all immunocompetent humans and Old World monkeys, administered *α*-gal epitopes form *in situ* immune complexes with it. One area demonstrating this Ag/Ab interaction has been xenotransplantation in which pig cells or pig organs are transplanted into humans or monkeys. Anti-Gal binding to the multiple *α*-gal epitopes on cells of pig xenografts causes the rapid rejection of such xenografts (e.g., pig heart or kidney) in humans, or in monkeys, by complement-dependent cytotoxicity (CDC) and by antibody-dependent cell cytotoxicity (ADCC) [42–45]. As described below, injection of *α*-gal glycolipids into tumors in a mouse experimental model producing anti-Gal Ab results in expression of *α*-gal epitopes on tumor cells within the injected lesion, in a manner similar to the expression of these epitopes on pig cells. The subsequent binding of anti-Gal Ab to the *α*-gal epitopes *de novo* expressed on the tumor cells results in destruction of tumor cells as in xenograft rejection and the targeting of tumor cells and cell membranes to APC.

## 4. Anti-Gal-Mediated Targeting of Ags to APC and Increased Immunogenicity in the OVA model

The principle of anti-Gal-mediated increased immunogenicity of vaccines by formation of immune complexes with *α*-gal epitopes could be illustrated with hen egg ovalbumin (OVA) as the immunizing Ag [46]. OVA serves as an effective model for an immunizing Ag since there are several highly sensitive immunological tools that enable evaluation of the internalization and processing of this Ag in APC and presentation of its most immunogenic peptide SIINFEKL on class I MHC molecules. Furthermore, activation of CD8+ T cells can be evaluated following the specific interaction with SIINFEKL when presented on class I MHC molecules. The immune response was analyzed in the experimental model of *α*1,3GT knockout mice (KO mice) which lack *α*-gal epitopes and produce the anti-Gal Ab [47]. OVA was encapsulated within liposomes that express multiple *α*-gal epitopes (referred to as *α*-gal liposomes) [46, 48, 49]. Uptaking and processing of OVA encapsulated within *α*-gal liposomes by KO mouse APC was found to be several fold higher when the liposomes bound anti-Gal than in the absence of this Ab. This increased uptake was due to Fc/Fc*γ*R interaction between the Fc portion of anti-Gal on liposomes and Fc*γ*R on APC [46]. APC in draining lymph nodes displayed 5–8 fold higher presentation of SIINFEKL than APC in draining lymph nodes of mice lacking anti-Gal. Accordingly, the activation of SIINFEKL specific T cells as measured by intracellular staining for IFN*γ* production and by binding of tetramers carrying SIINFEKL was 2–6 fold higher in the presence of anti-Gal in the immunized mice than in the absence of this targeting Ab [46]. In addition, cytolytic activity of SIINFEKL-specific T cells was ~8 fold higher and the titer of anti-OVA Abs was 32 fold higher in vaccinated mice that had the anti-Gal Ab than in mice lacking this Ab. These studies confirmed the hypothesis that anti-Gal binding to vaccinating Ags presenting *α*-gal epitopes induces effective uptake and processing of the Ag by APC and increased transport to draining lymph nodes. In the lymph nodes the immunogenic peptides presented by APC induce a markedly higher activation of CD8+ and CD4+ T cells than in the absence of this mechanism [46]. This conclusion is further supported by observations on the immune response to influenza virus vaccine and to gp120 of HIV following anti-Gal-mediated targeting to APC. Inactivated influenza virus processed enzymatically to express *α*-gal epitopes elicited a 100-fold higher specific CD4+ T cell response and anti-influenza Ab response and ~5 fold higher CD8+ T cell response than vaccinating virus lacking *α*-gal epitopes [50]. Accordingly, KO mice immunized with virus vaccine presenting *α*-gal epitopes were ~11 fold more resistant to challenge with live virus than KO mice immunized with virus lacking *α*-gal epitopes [50]. Similarly, gp120 envelop glycoprotein of HIV processed to express *α*-gal epitopes was found to elicit in KO mice ~100 fold higher T cell response (determined by ELISPOT) and anti-gp120 Ab response (determined by ELISA) than gp120 lacking *α*-gal epitopes [51]. All these studies in KO mice suggested that manipulation of autologous tumor cells in cancer patients to express *α*-gal epitopes may result in conversion of such cells into effective vaccines that elicit a protective immune response against autologous TAA because of their effective anti-Gal-mediated targeting to APC.

## 5. Increased Immunogenicity Following Anti-Gal-Mediated Uptake of Tumor Cells by APC 

Studies on increasing immunogenicity of TAA by anti-Gal-mediated targeting of tumor cells to APC were initially performed with the mouse melanoma B16 cell line, lacking *α*-gal epitopes, and processed to express these epitopes by stable transfection with the *α*1,3GT gene. These cells were irradiated and injected into anti-Gal producing KO mice [52–54]. Vaccination with irradiated B16 cells expressing *α*-gal epitopes was found to elicit a protective immune response that prevented tumor growth following challenge with live tumor cells lacking this epitope [52, 54]. Such protection was also found in mice challenged with live melanoma cells prior to the immunization with the irradiated tumor cells expressing the *α*-gal epitopes [53]. Recently, a similar protective effect of tumor cells expressing *α*-gal epitopes has been demonstrated in a pancreatic adenocarcinoma model in KO mice [55]. The increased immunogenicity of TAA in both melanoma and pancreatic adenocarcinoma models was shown to be associated with effective anti-Gal-mediated uptake of the vaccinating cells by APC [52–55]. 

The method of genetic manipulation by stable transfection with *α*1,3GT gene is effective in inducing *α*-gal epitope expression in cell lines since it requires cell division for the insertion of this gene into the DNA of the tumor cells. Because of the need for extensive cell division, this genetic manipulation is not effective *in vivo* in solid tumors that are injected with a vector containing the *α*1,3GT gene. Introduction of the *α*1,3GT gene into tumor cells and transient expression of *α*-gal epitopes was also found to be effective in cell lines using an adenovirus vector containing this gene [56]. Accordingly, immunization with B16 melanoma cells transduced with adenovirus containing the *α*1,3GT gene was found to elicit immune protection against the tumor challenge [56]. However, injection of this vector into tumor lesions resulted in a very limited expression of *α*-gal epitopes because of poor diffusion of the injected virus beyond the injection area (unpublished observations). An alternative method has been developed for *in vivo* expression of *α*-gal epitopes on autologous tumor cells which consists of intratumoral injection of *α*-gal glycolipids. 

## 6. ***α***-Gal Glycolipids Insert ***α***-gal Epitopes into the Membrane of Tumor Cells within Treated Lesions

**Figure 1:**
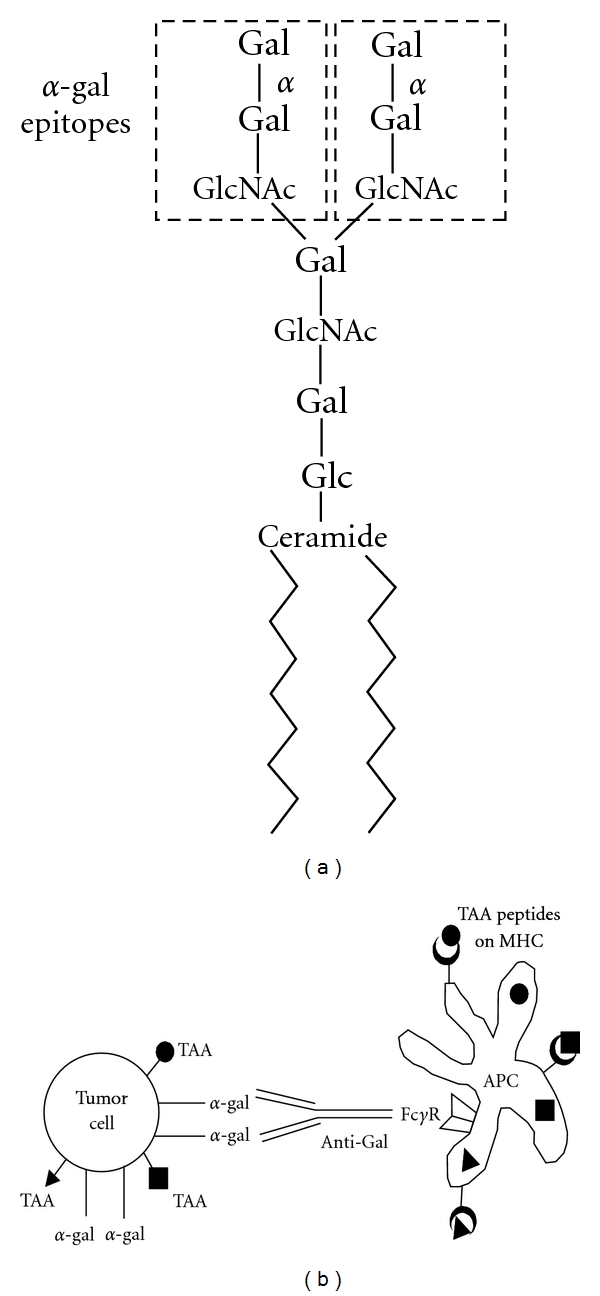
(a) Ceramide decahexoside as a representative *α*-gal glycolipid. This glycolipid has 10 carbohydrate branched chains. The *α*-gal epitope (Gal*α*1-3Gal*β*1-4GlcNAc-R) marked by the broken line rectangles caps both carbohydrate branches. The terminal *α*-galactosyl (Gal) unit is linked *α*1,3 to the penultimate Gal of the carbohydrate chain by the glycosylation enzyme *α*1,3galactosyltransferase (*α*1,3GT). The lipid portion of *α*-gal glycolipids (ceramide) anchors the carbohydrate portion in the cell membrane via the two fatty acid tails. (b) Anti-Gal-mediated targeting of tumor cells to APC in lesions injected with *α*-gal glycolipids. Intratumoral injection of *α*-gal glycolipids results in insertion of these glycolipids in tumor cell membranes. Anti-Gal IgG binds *in situ* to *α*-gal epitopes on the inserted glycolipids. Subsequent interaction between the Fc portion of the bound anti-Gal and Fc*γ*R on the APC (illustrated as a dendritic cell) induces uptake of intact or lysed tumor cells by APC, resulting in effective internalization of the tumor-associated antigens (TAAs). Internalized TAA are processed and various immunogenic TAA peptides (*⚫*, ■, ▲) are presented by the APC in association with class I and class II MHC molecules. These immunogenic peptides can activate tumor specific cytotoxic and helper T cells and elicit a protective antitumor immune response.

*α*-Gal glycolipids are glycolipids consisting of a ceramide lipid tail and a carbohydrate chain with one or several carbohydrate branches (antennae), all of which are capped with the *α*-gal epitope [57]. A representative *α*-gal glycolipid with 10 carbohydrate units (ceramide decahexoside) and two branches is illustrated in Figure 1(a). The number of branches carrying *α*-gal epitopes can be 1–5, or more, all having the *α*-gal epitope as the terminal carbohydrate structure. Rabbit red blood cells (RBCs) were found to be a very rich source of *α*-gal glycolipids. These RBC have on their membrane *α*-gal glycolipids with 5 carbohydrates and with longer chains that increase in size by increment of 5 carbohydrate (i.e., 10, 15, 20, and up to 40 carbohydrates (with the exception of a glycolipid with 7 carbohydrates)) [58–63]. Each increment also generates a new branch capped with *α*-gal epitopes [58–63]. *α*-Gal glycolipids are extracted from rabbit RBC membranes by their incubation in a mixture of chloroform and methanol [57]. The hydrophilic glycolipids are further separated from the hydrophobic phospholipids and cholesterol by a process called Folch partition [64]. Extracted *α*-gal glycolipids dissolve in water or PBS in a ball-like forms called micelles in which the hydrophobic portion of the glycolipid (i.e., the lipid tail) is in the core of the micelle whereas the hydrophilic carbohydrate chain protrudes into the aqueous surrounding. 

Incubation of *α*-gal glycolipid micelles with tumor cells for 2 h at 37°C results in spontaneous insertion of these glycolipids into the tumor cell membranes. This is since the hydrophobic lipid tail of the *α*-gal glycolipid is energetically much more stable when surrounded by the phospholipids of the lipid bilayer in the cell membrane than when it is surrounded by water molecules in the micelle. The insertion of *α*-gal glycolipids into the tumor cell membranes results in presentation of multiple *α*-gal epitopes on tumor cells. These epitopes protrude from the tumor cell membrane and readily bind the anti-Gal Ab (Figure 1(b)) [57]. The effective *in vitro* insertion *α*-gal glycolipids into B16 melanoma cells [57] and into human tumor cells [65] suggested that a similar insertion may occur *in vivo* in tumor lesions injected with these glycolipids. Effective *in vivo* insertion into a large proportion of the tumor cells within the lesion is achieved by injection in several regions of the tumor. It should be stressed that this insertion is not selective and occurs in both malignant and normal cells in the lesion. This *in vivo* insertion could be visualized in B16 melanoma lesions by staining with a lectin specific for *α*-gal epitopes (*Bandeiraea (Griffonia) simplicifolia IB_4_*) [57]. Injection of *α*-gal glycolipid micelles into tumor lesions is likely to result in several processes includingthe following.

### 6.1. Recruitment of APC by Complement Cleavage Chemotactic Factors

Anti-Gal/*α*-gal epitope interaction activates the complement system and generates chemotactic complement cleavage peptides such as C3a and C5a. These chemotactic factors induce an extensive recruitment of APC such as macrophages and dendritic cells into the treated lesion. Thus, this treatment enables the immune system to overcome the immunosuppressive conditions within solid tumor lesions, induced by microenvironment and local cytokine milieu and by regulatory T (Treg) cells [17–19, 65].

### 6.2. Insertion of *α*-gal Glycolipids into Tumor Cell Membranes

*α*-Gal glycolipids injected as micelles into lesions spontaneously insert into the tumor cell membranes, resulting in the presentation of multiple *α*-gal epitopes on the membrane of the tumor cells (Figure 1(b)).

### 6.3. Destruction of Tumor Cells by Anti-Gal Binding to *α*-gal Epitopes on the Cells

Anti-Gal binding to *α*-gal epitopes of the inserted glycolipids mediates tumor cell destruction in a process similar to xenograft rejection. Bound anti-Gal IgM molecules activate complement and induce cell lysis by complement-dependent cytotoxicity (CDC) [57]. Binding of anti-Gal IgG molecules to *α*-gal epitopes on cells further facilitates Ab-dependent cell-mediated cytolysis of the cells (ADCC) [42].

### 6.4. Targeting of Tumor Cells to APC for Their Conversion into Endogenous TAA Vaccine

Anti-Gal IgG molecules bound to *α*-gal epitopes on tumor cells in treated lesions further bind via their Fc portion to Fc*γ*R on dendritic cells and macrophages and stimulate these APC to internalize the opsonized tumor cells and cell membranes with the autologous TAA (Figure 1(b)). The internalized TAA, transported by the APC to draining lymph nodes, are processed and the immunogenic TAA peptides presented for the activation of tumor-specific T cells. These activated T cells leave the lymph nodes a circulate in order to seek and destroy tumor cells in micrometastases which express the immunizing TAA.

## 7. Treatment of Mouse Melanoma by Intratumoral Injection of ***α***-gal Glycolipids Prevents Tumor Growth

Evaluation of *α*-gal glycolipid immunotherapy was performed in the model of KO-mice-bearing syngeneic cutaneous B16 melanoma [57, 65]. Melanoma lesions with a size of ~5 mm were formed within one week after subcutaneous injection of 10^6^ melanoma cells of the cell line B16. Injection of 1 mg *α*-gal glycolipids into such lesions resulted in *in situ* insertion of these glycolipids into tumor cell membranes which could be demonstrated by immunostaining of tumor sections with *Bandeiraea simplicifolia* IB4 lectin which binds specifically to *α*-gal epitopes [57]. The interaction between *α*-gal glycolipids injected into B16 melanoma lesions of KO mice and the anti-Gal Ab further resulted in activation of the complement system and the formation of complement cleavage chemotactic factors that induced rapid recruitment of dendritic cells and macrophages. Thus, effective recruitment could be demonstrated already within 48 h after injection [57]. This recruitment further increases within 7 days, but it could not be observed in PBS-injected tumors, implying that in the absence of *α*-gal glycolipids, the immune system is “oblivious” to the growing tumor [57]. A similar rapid recruitment of macrophages was observed in KO mouse skin after injection of *α*-gal nanoparticles (submicroscopic liposomes) comprised of *α*-gal glycolipids, phospholipids, and cholesterol [49]. This recruitment was inhibited by cobra venom factor which blocks activation of the complement system thereby preventing generation of complement chemotactic factors [49]. 

B16 melanoma is a very aggressive tumor that usually doubles its size in KO mouse skin every 4–8 days. However, the majority (65%) of melanoma lesions injected with *α*-gal glycolipids displayed no additional growth or regression in lesion size [57]. The remaining tumors displayed slower growth than control lesions injected with PBS (all of which did not stop growing). *In vitro* analysis of anti-Gal-mediated killing of B16 melanoma cells presenting *α*-gal epitopes indicated that both complement depended cytolysis and ADCC contribute to the destruction of tumor lesions injected [57]. A similar complement-mediated cytolysis by anti-Gal was observed in human melanoma cells that were incubated with *α*-gal glycolipids, then incubated with human serum containing anti-Gal Ab and complement [65].

## 8. Melanoma Lesions Injected with ***α***-gal Glycolipids Are Converted into Autologous Vaccines

Similar to the lack of immune response against tumors in patients with advanced disease, there is no protective immune response in KO mice against untreated B16 tumors. This is indicated by the complete lack of APC infiltration in B16 melanoma tumors injected with PBS [57]. Moreover, melanoma lesions ablated by intratumoral injection of ethanol elicit no protective immune response against distant untreated lesions [65]. However, intratumoral injection of *α*-gal glycolipids converts the treated lesion into an endogenous autologous tumor vaccine which elicits an immune response against autologous melanoma-associated antigens (MAAs) on the tumor cells. By using B16 melanoma cells producing OVA as a surrogate TAA and employing detection methods as those described above for *α*-gal liposomes containing OVA [46], it was possible to demonstrate effective *in vivo* uptake of the tumor cells by APC in lesions injected with *α*-gal glycolipids [57]. In addition, the subsequent transport and presentation of SIINFEKL (the immunodominant OVA peptide) in the draining lymph nodes was much higher in mice with *α*-gal glycolipid-injected tumors than in those with PBS-injected tumors, that is, the number of SIINFEKL presenting APC in the draining lymph node in the former group was much higher than in the latter. This, in turn, resulted in a much higher number of tumor-specific activated T cells which mediated a systemic protective antitumor immune response. These T cells could be demonstrated *in vitro* by determining the number of SIINFEKL-specific CD8+ T cells in mice with B16/OVA treated with *α*-gal glycolipids versus PBS and by demonstrating the marked increase in their cytolytic activity against cells presenting SIINFEKL on class I MHC molecules [57]. A similar increase in MAA-specific T cells was observed in B16-bearing mice treated with *α*-gal glycolipids versus PBS control by analysis of IFN*γ* secretion in ELISPOT following incubation with immunodominant MAA peptides of tyrosinase and gp100 [65]. 

The *in vivo *protective effect of *α*-gal glycolipids was determined by evaluation of distant tumor development after treatment. KO mice producing anti-Gal Ab and bearing B16 melanoma lesions received 3 weekly injections of 1 mg *α*-gal glycolipids. One day after the third injection, the mice were challenged in the contralateral flank with 5 × 10^5^ B16 cells and subsequent tumor growth was monitored. The majority (65%) of treated mice displayed no tumor growth in the challenge site whereas the remaining mice displayed a slower tumor growth than PBS-injected tumors [57]. Another control group consisted of mice in which the primary tumor was ablated by intratumoral injection of ethanol, similar to tumor ablation in the clinical setting. Ablation by ethanol successfully destroyed treated tumors, however this treatment did not induce any protective immune response against challenge with B16 cells in the contralateral flank [65]. 

Immunotherapy with *α*-gal glycolipids was further evaluated for inducing a protective immune response against an established distant micrometastasis. A micrometastasis was simulated by subcutaneous inoculation in the left flank with 10^4^B16 cells at the same time that the right flank was inoculated with 10^6^ tumor cells. After 5-6 days, the tumor developing in the right flank reached a size of 5 mm (“primary” tumor) whereas in the left flank inoculation site the tumor was not visible at that time point and simulated an established distant micrometastasis. The visible primary tumor received two intratumoral injections of either PBS or of *α*-gal glycolipids. In mice injected with PBS the simulated distant micrometastasis developed into a 4–12 mm lesion within 20 days. However, in 50% of mice in which primary tumors were injected with *α*-gal glycolipids, no lesions developed from the micrometastasis during the 30 days monitoring period. In the remaining mice, the simulated micrometastases developed, however, at a slower rate than that in the PBS-treated mice [65]. Overall, the prevention of tumor growth in the contralateral flank following injection of the primary tumor with *α*-gal glycolipids reflects the induction of a protective immune response against autologous MAA due to the conversion of the injected tumors into autologous vaccine. This further suggests that a similar treatment in humans may elicit an immune response capable of destroying micrometastases, thereby preventing them from developing into lethal metastases.

## 9. ***α***-Gal Glycolipid Treatment Activates Tumor-Specific CD8+ T Cells and Overcomes Regulatory T Cell Activity

Adoptive transfer studies were performed with spleen lymphocytes from tumor-bearing mice treated with *α*-gal glycolipids that were transferred into naïve KO mice. These studies aimed to identify the lymphocytes that mediate the protective immune response against the tumor. The recipients were inoculated subcutaneously with 5 × 10^5^ B16 cells. This inoculation was performed 24 h prior to the adoptive transfer of 40 × 10^6^ spleen lymphocytes from B16 tumor-bearing donors treated with *α*-gal glycolipids or with PBS. In naïve mice that did not receive transferred lymphocytes, the challenging tumor cells developed into 5–7 mm lesions within 10 days and into a 20 mm lesion within 20–25 days. Adoptive transfer of lymphocytes from tumor-bearing mice treated with *α*-gal glycolipids resulted in prevention of tumor growth in ~70% of the recipients and the remaining recipients displayed slower tumor growth than in control mice [65]. However, when the transferred lymphocytes were depleted *in vitro* of CD8+ T cells (by anti-CD8-coated magnetic microbeads), the protective effect of the transferred lymphocytes was eliminated [65]. 

Lymphocytes transferred from mice with PBS-treated tumors had almost no protective effect and tumor growth was observed in >75% of the recipients [65]. Nevertheless, depletion of CD4+ T cells from the transferred lymphocytes resulted in increased protection against the tumor challenge [65]. These findings suggest that, in accordance with previous reports [66, 67], mice bearing B16 melanoma or other tumors have CD4+ regulatory T (Treg) cells that inhibit the development of a protective antitumor immune response. Thus, treatment with *α*-gal glycolipids seems to elicit a protective immune response potent enough to overcome the suppressive effect of endogenous Treg in the tumor-bearing mice [65].

## 10. Feasibility of ***α***-Gal Glycolipid in Immunotherapy of Cancer Patients

In studies in humans, anti-Gal from human serum was found to induce effective targeting of tumor cells presenting *α*-gal epitopes for uptake by human macrophages and dendritic cells via Fc/Fc*γ*R interaction [68]. The *in vivo* safety of such targeting by *α*-gal glycolipid immunotherapy has been evaluated in a Phase I clinical trial under IND-12946 at UMass Medical Center in patients with advanced solid tumors. Patients with a variety of advanced cancers received a single intratumoral injection of *α*-gal glycolipid into one of their tumor lesions. The administration of *α*-gal glycolipids was performed by endoscopy, laparoscopy, or ultrasound guidance, depending on the site of the tumor. Using standard Phase I dose escalation, each cohort of patients received an intratumoral injection of 0.1, 1.0, or 10 mg *α*-gal glycolipids. All participating patients displayed no treatment-associated toxicity (manuscript in preparation). Based on these studies, the dose of *α*-gal glycolipids to be used in future Phase II studies is planned to be 10 mg. It is expected that the results of *α*-gal glycolipid treatment will vary from one patient to the other, depending on the immunogenicity of the various TAA in the individual patient and on the potency of the immune system in the treated patient. It is possible that in a proportion of the patients, the combination of effective TAA and potent immune system may result in the generation of a protective immune response against the autologous TAA that is effective enough to destroy tumor cells presenting these TAA. 

The studies in mice strongly suggest that the elicited immune response against autologous TAA is potent enough to destroy small groups of tumor cells, that is, micrometastases. However, it is not clear at present whether the *α*-gal glycolipid immunotherapy can elicit an immune response capable of destroying visible metastases. Studies in mice with B16 melanoma lesions [52] have demonstrated the effective destruction of the outer regions of visible tumors whereas the inner regions are not affected because of the inability of the T cells to infiltrate the core of such metastases. In view of the recent advances in immunotherapy by nonspecific modulators of the immune system such as the monoclonal Ab ipilimumab (anti-CTLA4 Ab enhancing T cell response), it may be possible that combination of such treatments with *α*-gal glycolipid treatment will have a synergistic immunoprotective effect. This is since the generation of activated T cells with autologous TAA specificity following the *α*-gal glycolipid treatment will be greatly enhanced by the subsequent “nonspecific” activation of the immune system by immunomodulators. Thus, it is possible that such a combination may induce a protective antitumor immune response that effectively destroys both micrometastases and visible metastases.

In addition to the possible use of *α*-gal glycolipids in immunotherapy of patients with advanced tumor, this treatment may be beneficial in improving prognosis when used as part of neoadjuvant immunotherapy in high-risk patients, prior to the resection of the primary tumor. One specific example may be mammary carcinoma. In a substantial proportion of women with mammary carcinoma, tumors originating from micrometastases reappear months to years after the resection of the primary tumor. In the suggested therapy, the primary mammary tumor is injected with *α*-gal glycolipids immediately after detection. The tumor is resected 3-4 weeks after this injection, as part of the standard clinical care. This time frame will provide a sufficient period for the APC to internalize autologous TAA, transport them to the draining lymph nodes, and activate the immune system to react against tumor cells expressing these TAA. Thus, long after the resection of the primary tumor, the immune system may be able to continue detecting and destroying tumor cells within micrometastases, thereby preventing the development of lethal metastases.

## 11. Conclusions

A protective immune response against tumors can be achieved by activating the immune system to react against the full range of autologous tumor-associated antigens (TAAs). Many of these TAA differ from one cancer patient to the other and are formed by various mutations due to genomic instability. Presently, it is difficult to identify the multiple autologous TAA in each patient in order to synthesize the various TAA peptides for vaccine preparation. Therefore, the tumor itself may serve as a source for the vaccinating TAA. In order for the tumor to function as a vaccine, tumor cells and cell membranes have to be effectively targeted to antigen-presenting cells (APCs) which process the TAA, transport them to the draining lymph nodes, and present the immunogenic TAA peptides for the activation of tumor-specific T cells. Tumor cells usually evolve to be “ignored” by APC and to develop without eliciting an antitumor immune response. Immunogenicity of tumors can be increased by manipulating them to express *α*-gal epitopes (Gal*α*1-3Gal*β*1-4GlcNAc-R). Injection of glycolipids with *α*-gal epitopes (*α*-gal glycolipids) in the form of micelles into tumors results in spontaneous insertion of the lipid tail of these glycolipids into the cell membrane and the presentation of multiple *α*-gal epitopes on the tumor cell membranes. This results in binding of the natural anti-Gal antibody (constituting 1% of immunoglobulins in humans) to its ligand, the *α*-gal epitope on tumor cells within the treated lesion. Anti-Gal opsonizes the tumor cells and targets them to APC via the interaction between the Fc portion of the bound anti-Gal and Fc*γ* receptors on APC. Such interaction induces effective uptake of the opsonized tumor cells by APC, and subsequent processing and presentation of TAA peptides. The elicited immune response is potent enough to overcome the immunosuppressive effect of regulatory T cells and to activate tumor-specific T cells which can destroy tumor cells within micrometastases. A phase I study (IND 12946) in patients with advanced solid tumor has indicated that intratumoral injection of 0.1, 1.0, and 10 mg *α*-gal glycolipids has no adverse effects. This immunotherapy aims to destroy micrometastases in cancer patients with advance disease. In addition, injection of *α*-gal glycolipids into primary tumors few weeks prior to resection may convert the lesion into a temporary autologous tumor vaccine which induces a protective immune response that will destroy micrometastases, long after the primary tumor has been resected.
